# MRI-based insights into brain structural and functional alterations in schizophrenia treated with risperidone

**DOI:** 10.3389/fpsyt.2025.1590886

**Published:** 2025-06-20

**Authors:** Hangyu Li, Xinyue Chen, Shiji Peng, Ying Li, Rui Yu, Kaike Liao, Nian Liu

**Affiliations:** Department of Radiology, Affiliated Hospital of North Sichuan Medical College, Nanchong, China

**Keywords:** magnetic resonance imaging, gray matter, white matter, fMRI, schizophrenia, risperidone, cognitive function

## Abstract

**Background:**

Schizophrenia (SZ) is a severe psychiatric disorder, with antipsychotics serving as the primary treatment. Among them, risperidone plays a crucial role in alleviating both positive and negative symptoms while also enhancing cognitive function. Advances in magnetic resonance imaging (MRI) technology have provided an effective means of investigating the effects of risperidone on the brain, particularly in terms of neural pathways, therapeutic efficacy, and predictive outcomes. This review offers a summary of current findings on the impact of risperidone treatment on gray matter, white matter, and functional brain activity and connectivity in SZ patients, including its neural mechanisms, therapeutic benefits, and potential side effects.

**Methods:**

Literatures on the use of risperidone for treating schizophrenia were searched in PubMed, Embase and Web of Science analyzing and summarizing the alterations in brain structure and function associated with risperidone.

**Results:**

Through the analysis and summary, it was found that risperidone treatment in SZ patients can have a marked effect on different structural and functional regions including the prefrontal lobe, temporal lobe, cingulate gyrus, corona radiata, basal ganglia, and corpus callosum.

**Conclusion:**

Most research has focused on short-term effects, with limited longitudinal data to assess long-term efficacy and side effects, more researches could be added in the future. In addition, more potential methods such as DKI, DSI and brain covariance network have the opportunity to be used in the study of brain structure and function in the treatment of schizophrenia with risperidone in the future.

## 1 Introduction

Schizophrenia (SZ) affects over 23 million people worldwide, with a lifetime prevalence of approximately 0.7% (1). In 2019, SZ ranked as the 20th leading cause of disability in the world (2) and became the ninth leading cause of disease burden in China (3). Characterized by cognitive impairment together with positive and negative symptoms, SZ presents significant challenges across the physical, psychological, and social domains. Treatment primarily involves antipsychotic medication, often complemented by psychological support and cognitive training, all of which are essential for optimizing patient quality of life and social functioning (4). Many SZ patients require lifelong antipsychotic therapy to maintain symptom stability (5). However, the long-term effects of these medications on the brain remain incompletely understood, underscoring the importance of selecting appropriate antipsychotics and evaluating their impact—both therapeutic and adverse—on brain structure and function.

The advent of atypical antipsychotics has revolutionized SZ treatment, and they now form the cornerstone of disease management (6). Unlike typical antipsychotics, which primarily target positive symptoms, atypical antipsychotics also alleviate negative symptoms and cognitive deficits while exhibiting lower rates of side effects (7). Among them, risperidone is one of the most widely prescribed agents due to its demonstrated efficacy in SZ management (8). Relative to typical antipsychotics including chlorpromazine, risperidone is more effective in terms of its ability to improve negative symptoms and cognitive function (9) and is associated with fewer extrapyramidal side effects (7). Consequently, risperidone is frequently used as a first-line treatment for both acute and chronic SZ (10, 11). However, its use is not without drawbacks, particularly its association with an increased risk of metabolic syndrome (MetS) (12). MetS, which entails a cluster of metabolic and clinical abnormalities, significantly elevates the risk of stroke, type 2 diabetes, and coronary artery disease (13). The prevalence of MetS is markedly higher among SZ patients receiving long-term antipsychotic therapy (38.1%) compared to those untreated (12.3%) (14), contributing to increased mortality rates in this population (15). Advancements in MRI technology have enabled researchers to explore the effects of risperidone on the brain in SZ patients, with a particular focus on neural pathway mechanisms, treatment efficacy, and predictive markers. This review provides a comprehensive analysis of risperidone’s impact on brain structure and function, including alterations in gray matter, white matter, and functional connectivity, as well as its therapeutic effects and associated side effects (e.g., MetS). By integrating MRI-based findings, this review aims to enhance our understanding of risperidone’s role in modulating brain alterations in SZ, distinguishing between disease-related alterations and treatment-induced effects.

## 2 Searching procedures

This is a narrative review, and we searched PubMed, Embase, and Web of Science from January 1, 2005, to December 31, 2024, for publications. Keyword terms: “risperidone” and “MRI” or “magnetic resonance imaging” and “schizophrenia”. We also restricted the search to articles written in English.

Studies were included based on the following criteria (a) peer-reviewed primary literature (b) MRI studies (c) effects of risperidone treatment of SZ on the brain. Studies were excluded for any of the following reasons: (a) MRI has not been used as a primary method to study the neurological basis of SZ, (b) the effects of risperidone treatment on changes in the patient’s brain were not described (c) the patients studied had a disorder other than SZ, such as bipolar disorder, depression, transient psychotic disorders, delusional disorder, or a psychotic disorder that was not otherwise specified (d) antipsychotics Mixed treatment.

**Figure 1:**
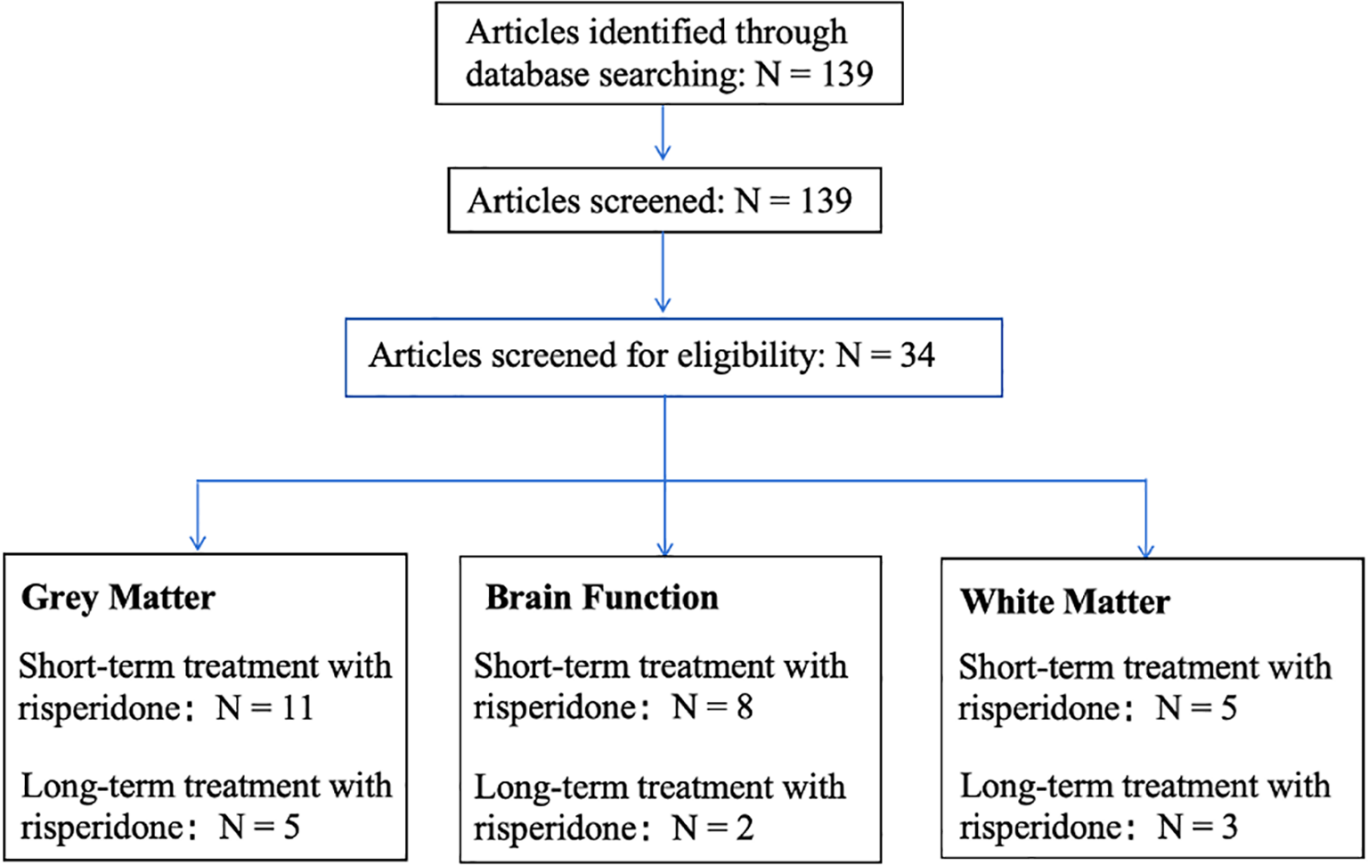
Search flowchart.

The flowchart is shown in **Figure 1**, of the 139 papers found by keyword search, 16 papers related to risperidone treatment of gray matter alterations in the brain in SZ met the inclusion criteria, including 11 for short-term treatment (Reference: 23-33) and 5 for long-term treatment (Reference: 34-38); 10 papers related to alterations in brain function met the inclusion criteria, including 8 for short-term treatment (Reference: 46-53) and 2 for long-term treatment (Reference: 54,55), and 8 papers related to alterations in white matter in the brain met the inclusion criteria, 5 short-term treatments (Reference: 59-63) and 3 long-term treatments (Reference: 64,65).

## 3 Neural pathways underlying the risperidone-mediated treatment of schizophrenia

Risperidone, an atypical antipsychotic, exerts its effects primarily through antagonism of D2 dopamine receptors, 5-HT2A/2C serotonin receptors, and α1/α2-adrenergic receptors (16). Its therapeutic efficacy in SZ stems from its ability to modulate both the dopaminergic and serotonergic systems. As a 5-HT2A/D2 receptor antagonist (17), risperidone plays a crucial role in alleviating psychiatric symptoms by blocking D2 receptors in both the nucleus accumbens and prefrontal cortex (18), thereby enhancing dopamine release in the striatum and prefrontal cortex (17). Risperidone mitigates positive symptoms including delusions and hallucinations via antagonizing D2 receptors in the mesolimbic pathway, thereby reducing excessive dopaminergic activity (19). Additionally, it can antagonize the 5-HT2A receptor to modulate cortico-striatal-thalamic circuits, with this being associated with improvements in negative symptoms and cognitive function (20). However, risperidone’s blockade of D2 receptors in the nigrostriatal pathway may lead to extrapyramidal symptoms, while its suppression of tuberoinfundibular pathway D2 receptors can result in hyperprolactinemia and associated sexual dysfunction (18), and risperidone’s antagonistic activity on α1/α2-adrenergic receptors has also been implicated in weight gain, metabolic disturbances, and sedation (21). And there is growing evidence that atypical antipsychotics, including risperidone, are more likely to lead to the development of the MetS (22, 23). MetS is highly prevalent in patients with SZ, with a prevalence twice that of the general population, and manifests itself in weight gain, dyslipidemia, type 2 diabetes, and hypertension (24), which together contribute to a significant reduction in life expectancy and treatment adherence (25, 26).

Overall, risperidone’s therapeutic effects are mediated through multiple neural pathways. It improves positive symptoms by antagonizing D2 receptors in the mesolimbic pathway, enhances dopamine release in the mesocortical pathway, and alleviates negative symptoms and cognitive deficits through 5-HT2A receptor modulation in the cortico-striatal-thalamic circuit. Additionally, MRI research suggests that risperidone may induce structural brain alterations, further contributing to its clinical efficacy in SZ and the development of the MetS. However, the effects of short-term and long-term use of risperidone on the brain of patient with SZ may be different, and the short-term benefits of risperidone treatment are well known, but the benefits of long-term use remain unclear, mainly because most clinical trials focus on short- or medium-term follow-up. Therefore, this review defines the long-term and short-term use of risperidone based on reference (27), with short-term treatment up to 2 years and long-term treatment with more than 2 years.

## 4 Gray matter alterations and associated clinical outcomes in risperidone-treated schizophrenia

### 4.1 Gray matter alterations and clinical outcomes associated with short-term risperidone treatment

Short-term risperidone treatment induces detectable alterations in gray matter in SZ patients, which may be linked to early improvements in clinical symptoms. Previous studies have demonstrated that short-term risperidone treatment is associated with increased gray matter volume in the basal ganglia (28), inferior parietal lobe (29), right prefrontal lobe (30), temporal lobe (29, 31), occipital lobe (32), parahippocampal gyrus (29), with the basal ganglia exhibiting the most pronounced alterations. Additionally, cortical thickness increases have been observed in the prefrontal lobe (33), whereas decreases in such thickness have been reported in the insula, superior temporal gyrus, and inferior parietal lobe (34, 35). Ebdruo et al. (28) found that patients with first-episode SZ presented with an increase in the gray matter volume in the caudate nucleus and nucleus accumbens following treatment with risperidone for three weeks, which correlated with cognitive improvements. In line with this observation, Zhang et al. (30, 31) found that six weeks of risperidone treatment led to an increase in gray matter volume in the left nucleus accumbens, thalamus, right prefrontal cortex, and supratemporal gyrus, with these alterations being associated with improvements in cognitive function and a reduction in positive symptoms. Gray matter alterations in SZ patients treated with risperidone for 3–6 weeks primarily localized to the caudate nucleus, nucleus accumbens, prefrontal cortex, and superior temporal gyrus. Notably, clozapine—an atypical antipsychotic with distinct pharmacological properties—demonstrated comparable but temporally extended effects: short-term administration (spanning 6-month to 2-year follow-ups) reduced gray matter volume in the caudate nucleus, nucleus accumbens, and prefrontal cortex, correlating with clinical symptom amelioration. This differential effect may be attributable to clozapine’s predominant use in treatment-refractory SZ populations, where severe neuropathology and prolonged illness duration could predispose to pronounced structural neuroadaptations.

However, findings regarding gray matter volume alterations following eight weeks of risperidone administration have been inconsistent. For instance, Goghari et al. (33) reported a significant increase in prefrontal cortical thickness alongside working memory improvements, whereas a separate study (36) observed reductions in prefrontal and temporal cortex thickness which were unrelated to clinical symptoms. This may be due to differences in their administration methods of risperidone - one involving titration and the other oral administration, combined with the younger age of patients in the former group. Additionally, the impact of schizophrenia progression on brain structural alterations should not be overlooked. These discrepancies may be attributed to individual variability, sample size inconsistencies, and inconsistent dosages, highlighting the complex biological mechanisms underlying risperidone treatment in SZ patients. Longer treatment durations further reveal distinct patterns of gray matter alterations. Massana et al. (37) showed that after three months of risperidone administration, first-episode SZ patients exhibited an increase in gray matter volume in both the caudate nucleus and nucleus ambiguus, with these alterations being correlated with cognitive improvement and in the alleviation of negative symptoms. Those patients who underwent treatment for one year demonstrated an increase in gray matter volume in the right parahippocampal gyrus, inferior temporal gyrus, occipital lobe, and subparietal lobule (29, 32), which was related to reductions in both positive and negative symptoms and enhanced working memory. However, thinning of the cotext in the insula and subparietal lobule was also evident (34, 35), though these alterations did not correlate with symptom improvement. In nodes of 8 weeks, 3 months, and 1 year of risperidone treatment for schizophrenia, changes occurred in cortical thickness and gray matter volume, mainly in the prefrontal, temporal, parietal, and occipital lobes.

**Figure 2:**
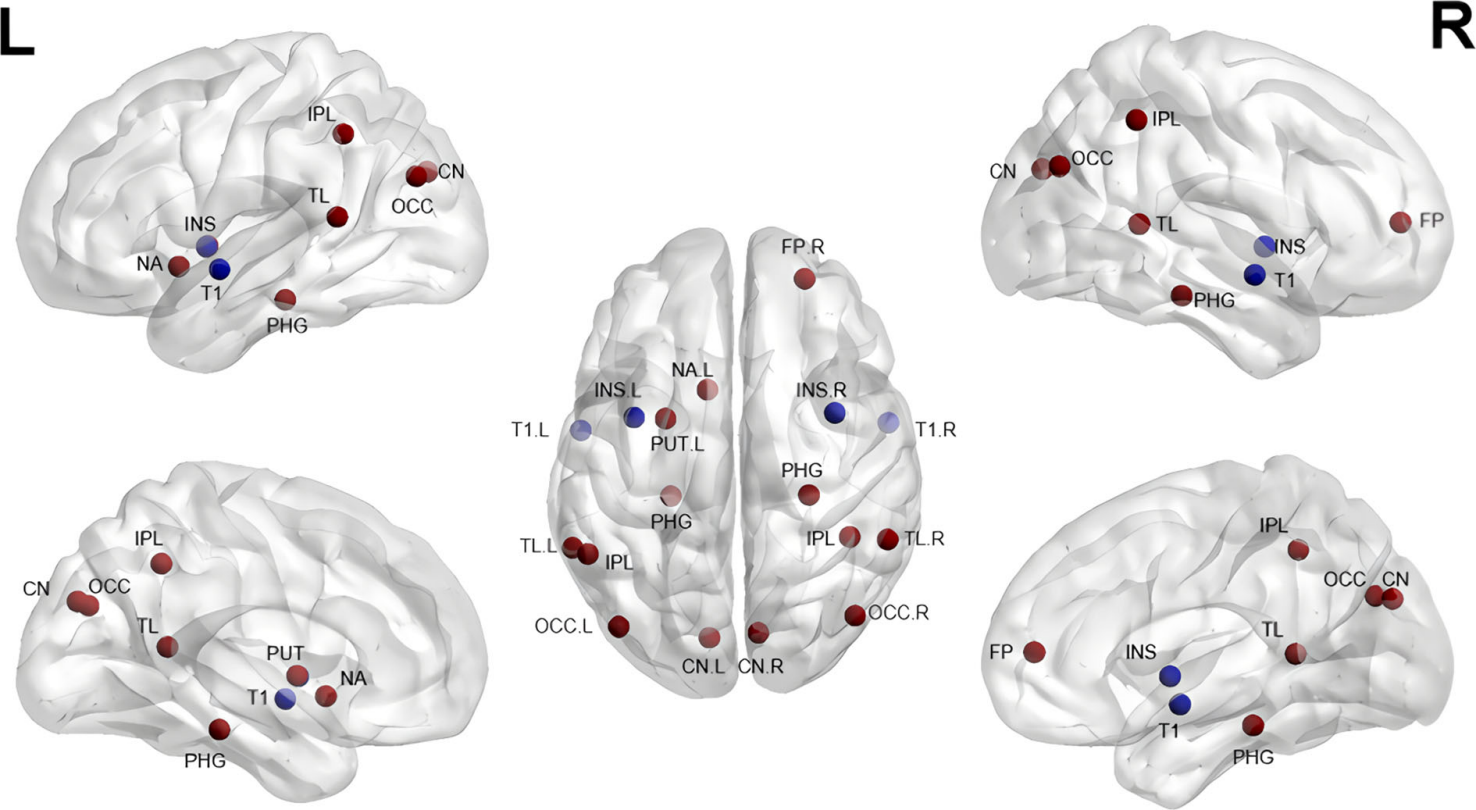
Gray matter alterations in short-term treatment of SZ with risperidone (References 28–38). CN, Caudate Nucleus; PUT, Putamen; NA, Nucleus Accumbens; INS, Insula; IPL, Inferior Parietal Lobule; PHG, Parahippocampal Gyrus; T1, Superior Temporal Gyrus; TL, Temporal Lobe; OCC, Occipital Lobe; FP, Frontal Pole. Red indicates an increase, blue a decrease.

In summary, short-term risperidone treatment is more frequently associated with increased gray matter volume in the frontotemporal lobe and basal ganglia (28, 38) (See **Figure 2** for details). However, the precise link between these structural brain alterations and clinical efficacy remains unclear. To overcome these issues, studies conducted in the future should aim to employ larger sample sizes while minimizing individual differences and controlling for variations in drug dosage. Additionally, while there is a close link between gray matter surface area and both neurodevelopment and psychiatric pathology, there have been few studies focused on this topic in SZ patients receiving risperidone. Investigating these alterations may provide a valuable means of bridging critical gaps in brain structure research and providing deeper insights into the mechanisms of antipsychotic efficacy, ultimately supporting the development of precision medicine.

### 4.2 Gray matter alterations and clinical outcomes associated with long-term risperidone treatment

Long-term treatment with risperidone in SZ patients leads to persistent gray matter alterations that have the potential to shape symptom management and prognosis. Among these alterations, gray matter volume reductions in the prefrontal, temporal, and inferior parietal lobule (39, 40) have emerged as hallmark alterations in SZ patients undergoing prolonged risperidone treatment. Yu et al. (39) performed a cross-sectional study demonstrating that reductions in gray matter volume in the prefrontal and temporal lobes were the predominant structural alterations evident in patients with chronic SZ treated with long-term risperidone. Similarly, our previous research (40) revealed bilateral decreases in gray matter volume in the prefrontal, temporal, and subparietal lobes in chronic SZ patients following prolonged risperidone treatment. Additionally, studies have reported gray matter volume reductions in the parietal lobe following prolonged antipsychotic treatment in SZ patients (41) and model animals (42, 43), with these being alterations not observed in studies of short-term treatment (29, 31, 33). This may indicate that reductions in the volume of parietal gray matter may arise specifically in response to prolonged antipsychotic exposure and could serve as a viable target for novel therapeutic strategies. However, as previous studies (39, 41) did not specifically examine parietal gray matter alterations following long-term risperidone treatment, further research is necessary to validate and expand upon these findings.

Overall, the poorly defined link between structural brain alterations and therapeutic efficacy in SZ patients undergoing risperidone treatment for extended periods may be influenced by several factors, such as individual variability, disease progression, and medication dosages. Further studies will be crucial to elucidate the long-term benefits of risperidone and its effects on the structure of the brain in SZ patients.

### 4.3 Gray matter alterations and treatment-related metabolic syndrome

Current evidence suggests a close link between alterations in the gray matter and MetS in SZ patients undergoing risperidone treatment. SZ patients with MetS have been found to present with abnormal structural and functional findings in the brain, such as reductions in overall brain volume, a decrease in regional volume and surface area, and dysregulated reward circuit integrity in the orbitofrontal cortex and insula, among other areas (44). Emsley et al. (45) found that among first-episode, unmedicated SZ patients, treatment with risperidone for 13 weeks led to decreased ventral mesencephalic volume, with these alterations being significantly linked to weight gain, bilateral reductions in HDL-cholesterol, and unilateral elevations in blood glucose levels. However, it remains uncertain as to whether alterations in the gray matter of SZ patients result from disease progression or are attributable to treatment-induced MetS. This uncertainty emphasizes an important focus of future research efforts seeking to aid in the management of the long-term benefits and risks of risperidone administration.

### 4.4 Gray matter alterations as predictors of therapeutic responses

Alterations in the gray matter may serve as potential biomarkers for predicting risperidone treatment responses in SZ patients. Molina et al. (46) revealed that cortical thickness could predict treatment efficacy in first-episode SZ patients, particularly in the frontal lobe, where risperidone was associated with cognitive improvements. In a similar vein, Cui et al. (47) applied machine learning techniques to detect structural features, including an increase in the gray matter volume in the right anterior cuneiform lobe, cuneate, and subparietal lobule, yielding an overall predictive accuracy of 69.68% (sensitivity: 83.96%; specificity: 72.41%). Additionally, Zong et al. (48) integrated multi-omics approaches—including subcortical connectivity covariance networks, transcriptomic features, and peripheral epigenetic modifications—to predict early risperidone treatment responses in SZ patients.

These findings highlight the potential utility of structural MRI-derived radiomic features as tools for predicting early treatment responses to risperidone through machine learning. However, the current predictive accuracy of these strategies is relatively low at present. Future research will need to focus on refining machine learning and deep learning techniques to improve the precision of treatment response predictions for risperidone and other antipsychotics in SZ patients. Furthermore, structural covariance networks can highlight the topology of brain structures, allowing one to study the nature of anatomical anomalies in the context of large-scale networks, rather than just regional variations, and may further provide additional valuable information for common analyses that consider voxels in isolation (49, 50). And it has emerged as a valuable analytical tool in treatment efficacy prediction. Through the incorporation of information regarding coordinated alterations across multiple regions of the brain, this approach has the potential to significantly improve the accuracy with which early treatment responses can be predicted. Future studies should further optimize these methods to improve prediction efficiency and provide novel insights for the individualized precision treatment of SZ.

## 5 Risperidone treatment-related alterations in brain function and associated clinical outcomes in schizophrenia

### 5.1 Alterations in brain function and clinical outcomes associated with short-term risperidone treatment

Short-term risperidone treatment in SZ patients induces significant alterations in brain functional activity and connectivity, which are closely associated with clinical outcomes. Following six weeks of risperidone treatment, SZ patients exhibited notable decreases in functional connectivity within the cingulate gyrus of the default mode network (DMN) (51), alongside reduced connectivity deficits in the dorsal attentional network (52), with both being inversely related to symptom severity. Additionally, an increase in the amplitude of low-frequency oscillations (ALFF) was observed in the frontal lobe, parietal lobe, left supratemporal gyrus, and right caudate nucleus, which correlated with significant clinical symptom improvement (53). While these results are intriguing, their inconsistency with subsequent findings emphasizes the complex issues that can arise when assessing the impact of risperidone on functional connectivity in the brain. For example, research exploring eight weeks of risperidone treatment in SZ patients has yielded inconsistent findings with respect to functional connectivity alterations compared to baseline. Zong et al. (54) and Wang et al. (55) determined that functional connectivity in the DMN significantly increased in the posterior cingulate gyrus/precuneus, medial prefrontal cortex, and right superior temporal gyrus, with corresponding alterations in the right medial middle frontal gyrus and left superior frontal gyrus. A positive correlation was noted between these increases and the improvement of positive symptoms. However, another study (56) determined that there was a significant decrease in functional connectivity in the right posterior cingulate gyrus and precuneus following eight weeks of risperidone treatment, with this reduction also being associated with improved positive symptoms. These results are in direct conflict with one another, possibly owing to patient-specific differences in treatment response, variability in sample characteristics, or methodological differences in functional connectivity analysis (See **Table 1** for details). Additional studies are needed to elucidate the underlying mechanistic basis for these findings. Moreover, the included studies exhibited heterogeneity in both patient cohorts (encompassing both first-episode drug-naïve individuals and those with relapsed SZ following medication discontinuation) and medication dosages. To minimize confounding effects from prior antipsychotic exposure, future investigations should prioritize standardized follow-up protocols focused exclusively on first-episode drug-naïve SZ populations. Further mechanistic studies are imperative to disentangle the neurobiological underpinnings of these observations, particularly regarding dose-dependent treatment effects and disease progression trajectories.

**Table 1:** Major alterations in brain function in short-term treatment of schizophrenia with risperidone.

Treatment Duration	Main Findings	Clinical Correlations
6-week treatment	• Increased ALFF in the frontal lobe, parietal lobe, left supratemporal gyrus, and right caudate nucleus • Decreased FC in the cingulate gyrus of the DMN	• ALFF increases positively correlated with clinical symptom improvement
8-week treatment	• Increased DMN FC in posterior cingulate gyrus/precuneus, medial prefrontal cortex, and right superior temporal gyrus (conflicting studies) • Decreased FC in right posterior cingulate gyrus/precuneus (contradictory findings)	Contradictory
2-month treatment	• Reduced local regional coherence and node clustering in the visual network • Reduced FC deficits between visual-somatomotor network and hippocampus-superior frontal gyrus	• Longitudinal alterations in visual cortex local regional coherence linked to general symptom improvement

ALFF, Amplitude of Low-Frequency Fluctuations; DMN, Default Mode Network; FC, Functional Connectivity.

Zhang et al. (57) conducted further exploration of these alterations and determined that following treatment with risperidone for two months, patients with SZ presented with a reduction in local regional coherence and node clustering within the visual network. Moreover, a reduction in connectivity deficits was noted between the visual-somatomotor network and the hippocampus-superior frontal gyrus, and a link was identified between improvements in general symptoms and longitudinal alterations in local coherence within the visual cortex. Following treatment for four months, patients demonstrated normalized aggregation coefficients and local network efficiencies, together with an increase in connectivity between key regions, including the right frontal pole and parietal lobule, the left inferior frontal gyrus and temporal pole, and the right middle temporal gyrus and left cuneate cortex, with all of these alterations being related to clinical symptom improvement (58). A majority of fMRI studies suggest that short-term treatment with risperidone primarily impacts the cingulate gyrus, cuneus, and frontal lobe. However, the precise relationship linking functional alterations in the brain to therapeutic efficacy has yet to be fully elucidated, and symptomatic improvement likely results from complex interactions between multiple factors. Future studies should leverage more refined and systematic experimental strategies to more fully document the interplay between functional brain alterations and treatment efficacy in SZ patients, offering deeper insights into the mechanisms underlying risperidone’s therapeutic effects.

### 5.2 Alterations in brain function and clinical outcomes associated with long-term risperidone treatment

Long-term risperidone use in chronic SZ patients results in specific regional decreases in brain functional activity and connectivity, potentially contributing to negative symptoms and disease progression. Animal models have been the primary focus of resting-state fMRI (rs-fMRI) studies exploring the long-term effects of risperidone on brain functionality, with studies in SZ patients remaining fairly rare, in part owing to the challenges associated with conducting long-term follow-ups in cases of polypharmacy. In those patients with chronic SZ patients who have undergone persistent risperidone treatment for over five years, reductions in functional connectivity were observed in the right fusiform gyrus, right inferior temporal gyrus, and right inferior occipital gyrus (59). Moreover, SZ patients with an average duration of disease exceeding 15 years exhibited an increase in local nodal degree and nodal efficiency in the frontal and parietal regions, alongside decreased nodal degree, nodal efficiency, and nodal local efficiency in the temporal lobe. Notably, the drop in nodal local efficiency was related to longer disease duration and more severe negative symptoms (60). A majority of rs-fMRI studies focused on the long-term impact of risperidone have examined animal models, whereas there has been limited research conducted in SZ patients. Future research should explore larger patient cohorts using both cross-sectional and longitudinal approaches to cultivate a more comprehensive understanding of the impact of risperidone on brain functional connectivity over time and how these alterations are related to therapeutic efficacy. Combining longitudinal follow-ups with animal models may help address inherent challenges and advance knowledge in this area.

### 5.3 Treatment-related functional alterations and metabolic syndrome

Alterations in brain functional connectivity induced by risperidone treatment may be closely linked to the onset of MetS, highlighting the possibility of a bidirectional interaction between them. An rs-fMRI study (61) revealed that SZ patients with MetS exhibited reduced perfusion in the left orbitofrontal cortex together with an increase in the functional connectivity evident within the left insula, left frontal plexus, and middle/superior frontal gyrus, with a significant negative correlation between abdominal obesity and perfusion in the left orbitofrontal cortex relative to those SZ patients unaffected by MetS. A separate rs-fMRI study (62) demonstrated the enhancement of functional connectivity between the cerebellum and the bilateral middle frontal gyrus and precuneus when analyzing those SZ patients with comorbid MetS relative to those unaffected by this metabolic condition. In a verbal working memory task, MetS patients also presented with a drop in blood oxygen level-dependent responses in the right superior frontal gyrus, right superior parietal gyrus, and left inferior parietal gyrus relative to healthy subjects (63). In addition, our recent study (15) found that (Regional homogeneity) in the right inferior orbitofrontal gyrus were significantly decreased and significantly negatively correlated with waist circumference, hip circumference, and BMI in SZ patients comorbid with MetS compared with those comorbid without MetS. These results emphasize the utility of fMRI as a means of clarifying the effects of MetS on brain function, particularly the relationship linking abdominal obesity to brain perfusion. The enhancement of perfusion and functional connectivity within specific regions of the brain-specific brain regions may facilitate the development of more effective, personalized treatment strategies. Future research should aim to clarify how metabolic interventions affect cognitive function and quality of life, aiming to develop comprehensive interventional strategies capable of improving overall prognostic outcomes in SZ patients with comorbid MetS.

## 6 White matter alterations and associated clinical outcomes in risperidone-treated schizophrenia

### 6.1 White matter alterations and clinical outcomes associated with short-term risperidone treatment

White matter alterations serve as key indicators for assessing treatment efficacy in SZ. Short-term risperidone treatment has been linked to decreased fractional anisotropy (FA) in the corona radiata (64, 65), prefrontal lobe (65), and corpus callosum (64). However, research examining first-episode, unmedicated SZ patients after six weeks of risperidone treatment has yielded inconsistent findings. Kraguljac et al. (66) did not detect any significantly altered diffusion indices following six weeks of treatment relative to pre-treatment levels. However, two other studies (64, 65) noted FA reductions in multiple white matter regions, including the bilateral posterior corona radiata, posterior limb of the left internal capsule, superior corona radiata, anterior corona radiata, posterior thalamic radiations, middle corpus callosum, and bilateral anterior cingulate fasciculus. Although both studies identified FA decreases in the anterior corona radiata, their conclusions diverged: one study (64) reported a correlation between FA reductions and clinical symptom improvement, whereas the other (65) detected no such relationship. These discrepancies may stem from individual variability, sample size differences, or variations in risperidone dosage, necessitating further investigation. Interestingly, these studies still had differences in the type of SZ patients, duration of illness, and medication dosage.

**Figure 3:**
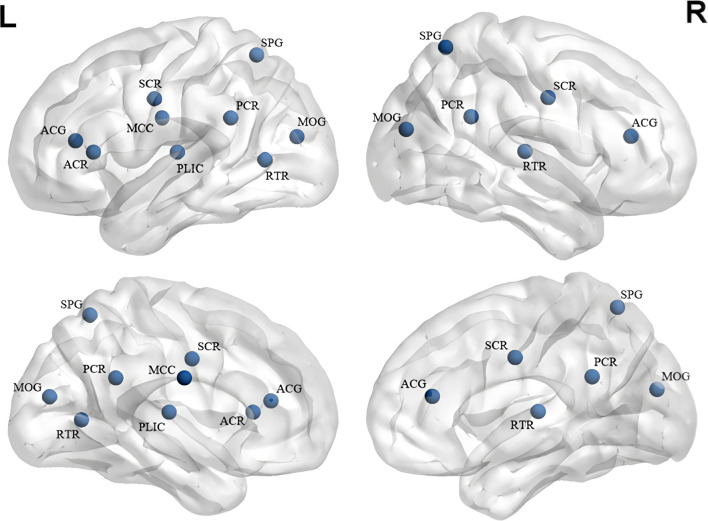
White matter alterations in short-term treatment of SZ with risperidone (References 64–68:). ACG, Anterior Cingulate Gyrus; ACR, Anterior Corona Radiata; MCC, Midbody of Corpus Callosum; SCR, Superior Corona Radiata; PLIC, Posterior Limb of Internal Capsule; PCR, Posterior Corona Radiata; SPG, Superior Parietal Gyrus; MOG, Middle Occipital Gyrus; RTR, Retrolenticular Thalamic Radiation. Blue represents a decrease in FA.

Hu et al. (67) determined that after eight weeks of risperidone administration, patients with first-episode SZ exhibited topographical improvements in the previously damaged limbic system for at least at one node, with these alterations being correlated with reductions in positive symptoms. However, no normalization of the prefrontal lobe’s topography was evident, nor were any alterations in negative symptoms documented. Szeszko et al. (68) reported FA decreases in the parietal and occipital white matter alongside increased radial diffusivity (RD) following 12 weeks of risperidone treatment, potentially linked to cognitive function improvements. Overall, short-term risperidone treatment in SZ patients primarily leads to FA reductions in the frontal lobe, corona radiata, and corpus callosum(See **Figure 3** for details).

Findings regarding white matter alterations after short-term risperidone treatment remain inconsistent, likely owing to individual differences, inconsistent treatment duration or dosing, and methodological variations. FA reductions observed during early treatment may be indicative of toxic side effects, including oxidative stress or excitatory neurotoxicity, or may indicate disease progression. While FA alterations have been associated with cognitive and clinical symptom improvements, the underlying mechanisms have yet to be clarified. Additional studies will be needed to determine whether FA alterations serve as reliable biomarkers of therapeutic efficacy.

### 6.2 White matter alterations and clinical outcomes associated with long-term risperidone treatment

Long-term treatment of SZ patients with risperidone leads to significant white matter alterations, though their relationship with clinical outcomes remains insufficiently explored owing to study limitations. Kahn et al. (69) found that SZ is predominantly characterized by the progressive time-dependent loss of gray matter, whereas damage to the white matter tends to be somewhat stable and can even show improvement with disease progression. In patients with chronic SZ undergoing prolonged treatment with risperidone, reduced FA is evident in multiple white matter regions, including the cingulate gyrus, the geniculate, somatic, and compressive portions of the corpus callosum, as well as the inferior and superior longitudinal fasciculi, external capsule, leptomeningeal fasciculus, posterior limb of the internal capsule, arcuate fasciculus, foramen, cerebellar peduncle, and corticospinal fasciculus (65). Compared to untreated patients, those receiving long-term risperidone treatment present with FA reductions in the left anterior thalamic radiation, left cingulate-hippocampal pathway, corpus callosum pressor, and geniculate regions, and left superior longitudinal fasciculus, whereas they present with greater FA reductions in the right leptomeningeal fasciculus (70).

Our previous study (71) revealed that prolonged treatment of chronic SZ with risperidone can result in alterations in the structural indices of the white matter network, including global topology, local node degree in the thalamus, prefrontal cortex, and occipital lobe regions, and reduced white matter connectivity. These shifts were found to predict cognitive function in addition to being positively correlated with cognitive performance, providing evidence that prolonged treatment with risperidone may aid in the preservation of white matter network integrity and contribute to cognitive improvements in SZ patients. White matter alterations related to long-term risperidone treatment predominantly affect regions including the corpus callosum and cingulate gyrus. Future studies should further investigate the long-term impact of risperidone on microstructural features in the white matter microstructure and their interplay with treatment efficacy to optimize therapeutic strategies and improve patient outcomes.

### 6.3 Gaps in current knowledge: the untapped potential of DKI and DSI

Diffusion tensor imaging (DTI) has yet to be implemented for the study of cerebral white matter alterations in SZ patients with comorbid MetS. However, DTI holds promise as a means of elucidating metabolic side effects through the detection of shifts in white matter structure and functional connectivity. Our recent study (72)based on DTI found that patients with SZ comorbid with MetS exhibited a reduction in bilateral thalamic degree centrality (DC) and nodal efficiency (NE), which were also associated with cognitive dysfunction and an increase in waist-to-hip ratio. When deployed in combination with advanced imaging techniques and metabolic metrics, DTI could aid in developing comprehensive models to enhance the current understanding of metabolic side effect-related brain responses. With the continued evolution of multidimensional approaches to SZ treatment, research in this area is expected to expand. Similarly, no studies have used diffusional kurtosis imaging (DKI) or diffusion spectrum imaging (DSI) to investigate white matter alterations in risperidone-treated SZ patients. These imaging modalities could help bridge existing research gaps. DKI is an extension of DTI that enables the quantification of non-Gaussian diffusion (73), thereby capturing the contribution of restricted diffusion in the measurement. Consequently, DKI offers additional complementary measures alongside standard DTI metrics (such as FA, MD, RD, etc.) that relate more closely to the underlying tissue microstructure (74). The ability of DSI to map complex fiber orientations has the potential to enhance current knowledge of white matter integrity. Wu et al. discovered (75)that the reduced structural integrity of the dorsolateral language pathway is associated with hallucinations in schizophrenia. Employing these advanced techniques may offer key insights and provide opportunities for more targeted and effective treatments for SZ patients with MetS.

## 7 Analysis of differences in brain alterations in patients with SZ treated with short-term risperidone therapy

Risperidone short-term treatment of patients with SZ showed differences in outcomes at 6 and 8 weeks, including gray matter, brain function, and white matter. The type of SZ patient, duration of illness, and dose of medication may be the main influencing factors. First, some studies were not first-episode drug-naïve SZ patient, and treatment with other antipsychotics can have a direct impact on the results. Second, as the course of SZ progresses, there can be significant effects on brain structure and function. Studies have found that patients with SZ treated with risperidone or clozapine for a long period of time show more areas of gray matter volume reduction compared to long-term untreated SZ. Therefore, it is hypothesized that risperidone or clozapine, while reducing symptoms and improving cognitive functioning in patients with SZ, may not be able to stop the pathophysiological process of SZ or counteract the process of progressive gray matter volume reduction in the brain. Finally, the dose of medication is an equally important factor. A meta-analysis found that less progressive gray matter loss in SZ patients treated with risperidone was associated with higher mean daily drug intake. Longitudinal studies of patients with first-episode SZ over a 14-year period also found that whole-brain gray matter volume reduction was associated with higher doses of antipsychotic medication. In summary, the type of SZ patient, duration of illness, and dose of medication may be key factors, and future studies will need to be controlled to investigate the specific role of risperidone or other antipsychotics.

## 8 Potential challenges and responses to long-term treatment of schizophrenia with antipsychotics

Because fewer SZ patients are taking one and the same antipsychotic medication for a long period of time, and because studies are limited by the uncontrollable nature of long follow-up, individual differences in disease progression, medication type and dosage, and socio-cognitive background. Therefore, there are fewer longitudinal follow-up studies on the effects of antipsychotics on brain alterations. However, exploring long-term monotherapy with antipsychotics is particularly important to study efficacy and side effects, and the following are some suggestions. First, include first-episode drug-naïve SZ patients to exclude the effects of other antipsychotic medication. Secondly, collaboration with psychiatric hospitals could be undertaken to centralize the management and medication of patients who meet the criteria. Finally, SZ patients should be scheduled for uniform MRI scans on a regular basis to standardize the examination process. Although long-term longitudinal follow-up is challenging, a deeper understanding of antipsychotic efficacy and side effects can be gained, providing some insights into the subsequent use of antipsychotics.

## 9 Conclusions and future directions

Risperidone treatment in SZ patients can have a marked effect on different regions of the brain including the prefrontal lobe, temporal lobe, cingulate gyrus, corona radiata, basal ganglia, and corpus callosum. Short-term treatment with risperidone brain alterations is mainly an increase in gray matter volume and a decrease in white matter FA drop, whereas long-term treatment will show a broader reduction in gray matter volume in more areas, and white matter may improve with long-term treatment. The difference in outcomes between short-term and long-term treatment may be due to the effects of disease duration and drug toxicity. Despite these findings, several limitations persist in current research. For one, small sample sizes have the potential to limit the generalizability and statistical power of findings. Secondly, most research has focused on short-term effects, with limited longitudinal data to assess long-term efficacy and metabolic side effects. Third, MRI alone is insufficient for directly assessing MetS, necessitating the integration of a range of clinical indices and biomarkers to support more comprehensive analysis.

To overcome these limitations, future studies should adopt large-scale, longitudinal designs integrating multimodal imaging approaches and biomarkers to systematically examine treatment-related brain alterations and therapeutic outcomes across different stages of SZ. Additionally, the relationship between white matter alterations and risperidone-associated side effects remains unexplored, highlighting a major research gap. Investigating this link could provide valuable insights into the underlying mechanisms connecting brain structural alterations with treatment-related risks. Furthermore, long-term assessments of risperidone’s efficacy and side effects are essential. Another promising avenue involves the development of predictive tools, including brain covariance networks or artificial intelligence models, as a means of estimating treatment efficacy based on structural and functional brain alterations. Moreover, research on the role of advanced imaging techniques, including DKI and DSI, in risperidone-treated SZ patients remains absent. Optimizing evaluation methods has the potential to enhance patients’ quality of life and facilitate more the management of treatment-related metabolic risks in a more effective manner.
